# Impact of Chip Breaker Geometry on the Performance of Actively Rotary Monolithic Turning Tools

**DOI:** 10.3390/ma18051154

**Published:** 2025-03-04

**Authors:** Richard Joch, Miroslav Cedzo, Andrej Czán, Michal Šajgalík, Jozef Holubják, Mário Drbúl, Jaromír Markovič, Miroslav Matuš

**Affiliations:** Department of Machining and Production Technology, Faculty of Mechanical Engineering, University of Žilina, Univerzitná 1, 010 26 Žilina, Slovakia; miroslav.cedzo@fstroj.uniza.sk (M.C.); andrej.czan@fstroj.uniza.sk (A.C.); michal.sajgalik@fstroj.uniza.sk (M.Š.); jozef.holubjak@fstroj.uniza.sk (J.H.); mario.drbul@fstroj.uniza.sk (M.D.); jaromir.markovic@fstroj.uniza.sk (J.M.); miroslav.matus@fstroj.uniza.sk (M.M.)

**Keywords:** chip formation, turning tool, machining

## Abstract

The control of chip formation is a key aspect of modern turning operations, as improper chip formation can negatively affect tool life, surface quality, and overall machining efficiency. One approach to improving chip control is the integration of a chip breaker into the geometry of the cutting tool. This study examines the impact of chip-breaking geometry on the performance of monolithic rotary tools in active rotation turning. Two types of tools were compared: one without a chip breaker and another with an integrated chip breaker. The functionality of the chip breaker was experimentally validated, demonstrating its effectiveness in shaping chip segmentation under specific process parameters. Furthermore, tool wear, workpiece surface roughness, and cutting forces were evaluated. The findings indicate that the tool equipped with a chip breaker exhibits reduced wear while maintaining comparable surface quality. However, this benefit is accompanied by a slight increase in cutting forces.

## 1. Introduction

In machining processes, the relative movement between the workpiece and the cutting tool is a fundamental determinant of machining performance. This relative motion in machining is a combination of primary and secondary movements. The material removal process is determined by these key movements [1]: the primary movement, also known as cutting speed (v_c_), and the secondary movement, referred to as the feed rate (f). The desired shape of the machined surface is achieved through the interaction of these movements, the tool geometry, and the positioning of the tool to the workpiece [2]. Turning is one of the most widely used machining technologies. In this process, the workpiece, clamped in the spindle, performs the primary rotational movement, while the cutting tool performs the feed motion. This coordinated movement enables both external and internal turning operations. Due to its versatility, turning applies to a wide range of materials and is regarded as one of the most efficient and cost-effective industrial machining processes. Furthermore, the excess material removed, in the form of chips, can be further recycled, significantly reducing production waste [3,4].

However, as the demand for enhanced functional properties and extended life of manufactured components increases, machining high-strength and hardened materials has become essential [5,6]. These materials generate significant forces and high temperatures during machining, leading to rapid tool wear and reducing tool life. As a result, productivity declines, and the overall quality of the manufacturing process is negatively impacted.

### 1.1. Rotary Tools

The high temperatures generated during machining influence both the mechanical and physical properties of the workpiece material and the cutting tool. Excessive heat accelerates tool wear, which is undesirable as it negatively impacts machining accuracy and reduces tool life. In metal machining, various methods are used to reduce heat generation and prolong the life of cutting tools. The most common approach is the application of cutting fluids [7,8,9]. However, due to their environmental impact, there is a growing shift toward reducing their use. An alternative solution involves using highly durable cutting tool materials, such as coated tools made of cubic boron nitride. Despite their advantages, these materials are limited by their thermal resistance and toughness, particularly when working with difficult-to-machine materials.

Among the new approaches to reducing heat build-up during turning is the implementation of a cooling cycle to minimize heat generation, similar to interrupted cutting. This concept involves either laterally shifting a broad tool that moves forward to the workpiece, allowing heat dissipation through the tool body, or using a rotating circular or non-circular cutting insert. The use of a non-circular cutting insert shows potential for machining non-circular profiles; however, achieving consistent results remains challenging due to the complexity of tool path adjustment [10]. In contrast, the use of a rotating circular cutting insert enables continuous cooling of the cutting edge. This machining kinematics demonstrates significant potential, particularly in extending the tool life, which is especially important due to the high costs of replacing cutting tools when turning hardened steels [11,12,13].

**Figure 1:**
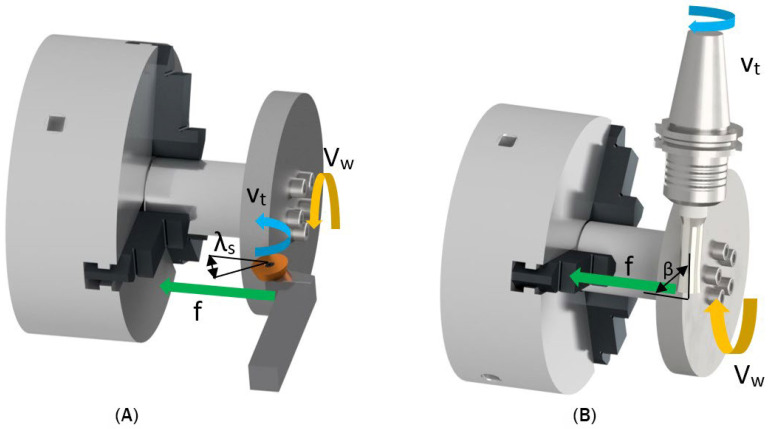
Scheme of rotary tools: (**A**) Self Propelled Rotary Tools; (**B**) Actively Driven Rotary Tools.

The circular tool continuously rotates around its axis during cutting, with the rotation of the tool distributing cutting energy along the entire circumference of the tool edge. Rotary tools can be classified into two types: Self Propelled Rotary Tools with bound rotation (SPRT) and Actively Driven Rotary Tools with active rotation (ADRT) [14,15,16]. In SPRT (Figure 1A), the rotational movement of the tool is driven by its interaction with the workpiece, whereas in ADRT (Figure 1B) an external drive system controls the speed of tool rotation [17,18]. The requirement for a rotating spindle, not typically found on conventional lathes, adds complexity and expense to the machining setup. This can be a significant barrier to adoption, especially for smaller workshops or those primarily focused on traditional turning. Additionally, the rotary tool itself can be more expensive than standard turning inserts.

During machining with rotary tools, three primary movements are defined: the workpiece rotation speed (v_w_), the tool rotation speed (v_t_), and the tool feed motion into the workpiece (f) [19,20]. Due to the rotational movement of the tool, the chip is drawn along the tool face. As a result, in orthogonal cutting, the chip speed deviates from its normal direction relative to the cutting edge. When machining with a tool with bound rotation, the rotation speed of the tool depends on the machining conditions, making it challenging to optimize the process [21]. On the other hand, a tool with active rotation has its rotation speed controlled by an external drive, allowing precise adjustment and enabling higher machining productivity. The drive system in ADRT technology enables the tool to regulate both rotation speed and direction (clockwise or counterclockwise). Among the parameters affecting tool speed, the tilt angle is the most significant and can be set to any desired value.

Compared to conventional turning, where the tool does not rotate, rotary turning strengthens the subsurface layer deeper into the workpiece. Turning with active rotation, all essential machining parameters, such as tool rotation direction, rotation speed, and tilt angle, can be precisely adjusted. When input parameters and conditions are properly set, machining time can be significantly reduced, and tool life can be extended. Although rotary tool turning is currently less commonly employed than conventional turning, its advantages become particularly evident when machining difficult-to-machine materials. As a result, its application is increasing and remains a focus of ongoing research.

### 1.2. Turning with Active Rotation

Despite numerous studies highlighting the potential of driven rotary tools, their application in real-world manufacturing processes remains limited due to several factors. Historically, these tools were developed for conventional machining, which proved inadequate due to low stiffness, limited flexibility, and insufficient productivity. Furthermore, these machines lacked the capability for programmable control of key parameters, including tilt angle, offset height, and tool rotation speed. As a result, further systematic research is required to advance the integration of driven rotary tools into practical production environments.

A driven rotary tool offers significantly higher stiffness and dynamic stability due to its innovative design. In addition, it enables precise adjustment not only of machining parameters but also the direction and speed of tool rotation, as well as the kinematic angles of intersection, independent of the workpiece [22]. Studies have shown that ADRT significantly extends tool life compared to conventional tools, particularly at higher cutting speeds and chip thicknesses [23]. Turning with active tool rotation allows for a wide range of adjustable machining parameters, including tool rotation direction, rotation speed, and engagement angle. As a result, this area presents extensive opportunities for further research.

Compared to conventional turning, both SPRT and ADRT tools have demonstrated significantly better performance in handling mechanical and thermal loads on the tool. Reported data indicate productivity gains of 400–500% and tool life extensions of up to 1500–2000%. The tool is capable of rotating in both clockwise and counterclockwise directions. Experimental results suggest that, when the tool rotates counterclockwise, it moves from the area where chips are formed to the area with fewer chips. Furthermore, surface imperfections increased with counterclockwise tool rotation and higher rotation speeds.

Harun et al. studied various parameters, including the effect of tool rotation speed (v_t_) on the surface roughness of the Ti-6Al-4V ELI alloy during turning with actively driven rotary tools [22]. Their findings indicated that an increase in tool rotation speed led to a decrease in surface roughness. However, this reduction was less significant compared to the impact of cutting speed and tool angle. A higher tool rotation speed can result in an increased dynamic tilt angle, causing a change in the chip flow direction and shifting the cutting mechanics from orthogonal to oblique cutting [24].

Sasahara et al. conducted turning experiments with active tool rotation without the use of cutting fluid, machining stainless steel SUS 304 and Inconel 718 [25,26]. Their findings indicate that, at a constant cutting speed, the peripheral temperature of the tool increases with the tilt angle. At a cutting speed of 100 m·min^−1^ and a tilt angle of 40°, a high peripheral tool temperature exceeding 400 °C was observed, along with significant adhesion on the tool surface. Across all tested cutting speeds, the peripheral temperature of the tool remained low at tilt angles of 10° and 20°. Beyond a tilt angle of 30°, a notable increase in chip temperature was observed. Tool wear after turning was measured at a cutting length of 70 m. No visible tool wear was detected at rotation speeds of 300 m·min^−1^ and 500 m·min^−1^. Noticeable tool wear was observed at a rotation speed of 100 m·min^−1^. When the tool remained stationary, significant tool wear was observed.

Hosokawa et al. studied the impact of tool and workpiece rotation speed on temperature. In contrast to previous studies, they measured the temperature on the flank face of the tool using infrared radiation and an optical fiber pyrometer. Their experiment maintained a constant cutting speed of 200 m·min^−1^ for the workpiece while varying its rotation speed from 0.005 m·min^−1^ to 300 m·min^−1^. The results indicated that the temperature at the cutting zone decreased as the tool rotation speed increased to approximately 250 m·min^−1^. However, beyond this speed, further increases in tool rotation caused a rise in cutting temperature. The observed temperature decrease at higher cutting speeds, up to a certain limit, was attributed to the cooling effect of the tool when outside the engagement zone.

Hosokawa [27] also identified issues related to chip segmentation during machining with a rotary tool and focused on chip segmentation during rotation. He proposed a chip breaker in the form of a spherical recess on the front face of the tool, which did not interfere with the cutting edge. The advantage of this solution is the uninterrupted cutting edge, resulting in continuous cutting. However, this solution may not be suitable for monolithic tools, which can be re-grinded after wear. Additionally, his findings indicated that the tool rotation direction had no measurable impact on cutting force or chip segmentation.

Research by Gürbüz et al. highlights the importance of chip breaker geometry on tool stresses and cutting forces during turning operations. Their findings indicate that different geometries can lead to varying chip-breaking efficiencies, which directly impact the overall machining performance [28]. Similarly, Cascón et al. developed a tailored chip breaker for polycrystalline diamond (PCD) inserts, demonstrating that a systematic design approach can effectively manage chip segmentation and improve surface quality during machining [29]. This aligns with the work of Li et al., who explored how chip breaker designs can enhance chip deformation and segmentation in drilling applications, suggesting that similar principles apply to turning [30]. Furthermore, the integration of chip breakers in actively driven rotary tools significantly enhanced chip control significantly. Umer et al. discussed the advantages of rotary tools, highlighting their ability to maintain continuous engagement of the cutting edge, which improves chip management and reduces the risk of chip entanglement [31]. This is particularly relevant in the context of difficult-to-machine materials, such as Inconel 718, where effective chip segmentation is essential for preserving surface integrity and extending tool life [32].

The study by Karabulut and Güllü further supports this, indicating that the position and design of chip breakers can effectively break long chips across various cutting conditions. The effectiveness of chip breakers is also influenced by both the cutting conditions and tool geometry. For instance, Słodki et al. demonstrated that high-pressure coolant delivery, combined with appropriate chip breaker geometry, can significantly improve chip segmentation and removal efficiency when machining titanium alloys [33]. This is further corroborated by Yang et al., who emphasized that chip breakers are essential in controlling chip flow, minimizing heat generation, and ultimately improving tool life and workpiece quality [34].

Despite numerous studies demonstrating the potential of turning with active tool rotation, this technology has not yet been widely implemented in real-world manufacturing processes for several reasons. First, ensuring the precise mounting of a replaceable cutting insert on the tool holder requires high accuracy. Secondly, standard cutting inserts and chip breakers are not designed for rotary tool movement. The design and application of chip breakers in actively driven rotary turning tools are important for optimizing machining processes. Thirdly, the geometry and placement of chip breakers can significantly influence the chip morphology, cutting forces, and overall machining efficiency. Therefore, future research should continue to explore innovative chip breaker designs tailored for various materials and machining conditions to enhance the performance of rotary turning tools.

## 2. Methodology

### 2.1. Monolithic Rotary Tool

In the turning process with active tool rotation, it is crucial to ensure sufficient stiffness of the machine-tool-workpiece system, stable tool clamping, and the elimination of clearance. Every cutting tool consists of a clamping section and a cutting section. Tools designed for turning with active rotation can be manufactured in two configurations. The first configuration consists of a tool assembly that includes a tool holder, a circular cutting insert, and a clamping screw [15,24,26,27,35]. The second configuration is a monolithic rotary tool, in which both the clamping and cutting sections are made from a single piece of material [36,37]. The use of a monolithic tool eliminates vibrations caused by inaccuracies in the positioning of the cutting insert, as well as the lower stability associated with securing the insert solely with a clamping screw. These factors contribute to the increased stiffness of the tool and the machine–tool–workpiece system.

The monolithic rotary tool used in this study is made of sintered carbide with a 10% cobalt content, ensuring high tool life and enabling the machining of hard and difficult-to-machine materials. The tool rake features a spherical surface, facilitating smooth chip evacuation from the cutting zone. Furthermore, the tool shank is tapered to simplify tool positioning to the workpiece and to prevent friction between the workpiece and the flank of the tool during machining.

The designed tool had a rake angle of γ = 8.2° and a clearance angle of α = 6.7°. The radius of the cutting edge was r = 0.0144 mm. The total length of the tool was 105 mm, with a cutting section diameter of 19 mm. The clamping section had a length of 50 mm and a shank diameter of 20 mm.

### 2.2. Chip Breaker on the Monolithic Rotary Tool

**Figure 2:**
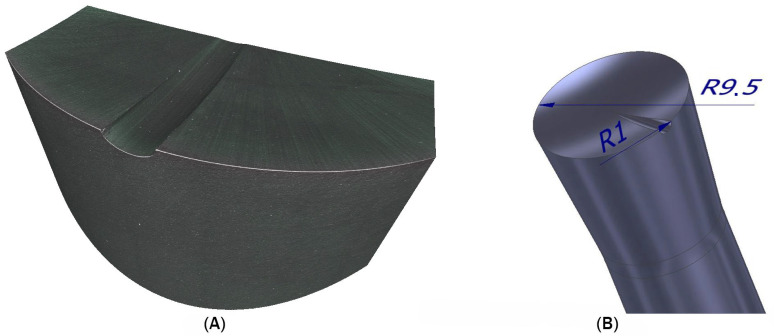
Chip breaker on the monolithic tool: (**A**) scanned image of tool; (**B**) model of tool.

In turning with active tool rotation, achieving the desired surface quality also requires proper chip-breaking. The dimensions and geometry of the tool are based on our previous scientific work and a patented monolithic tool solution. To ensure effective chip segmentation, a variant of the monolithic rotary tool with a chip breaker was designed. The circular rake face of the tool has a cutting section diameter identical to the monolithic tool without a chip breaker, measuring 19 mm. The ground chip breaker is semi-circular in shape with a diameter of 2 mm. Figure 2 presents a scanned image of the cutting section of the tool with the chip breaker, along with a model of the cutting section, including the dimensions of both the cutting edge and the chip breaker.

**Figure 3:**
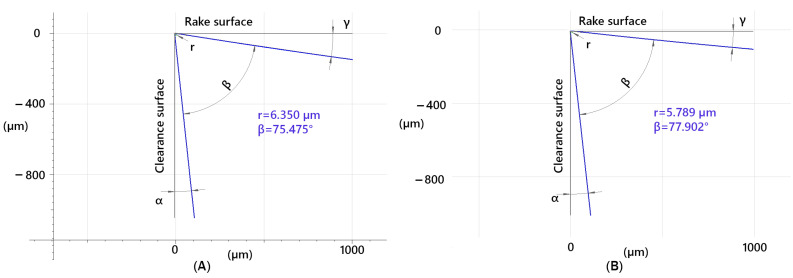
Tool geometry (**A**) with the chip breaker and (**B**) outside the chip breaker location.

The manufactured tool had slight variations in geometry at the chip breaker location compared to areas without it (Figure 3). Outside the chip breaker, the tool had a rake angle of γ = 8.4° and a clearance angle of α = 6.1°, with a cutting edge rounding radius of r = 6.35 µm. At the chip breaker location, the rake angle was γ = 5.8°, and the clearance angle was α = 6.3°, while the cutting edge rounding radius was r = 5.789 µm. TripleCoating Cr, which is applied using PVD technology, was selected for the construction of the tool with a chip breaker. This coating consists of an adhesive TiN layer, a middle AlTiN layer, and a top CrAlSiN layer. The coating combines the toughness and hardness of the middle AlTiN layer with the hardness of the nanocomposite CrAlSiN top layer. This coating is specifically designed for machining hardened and corrosion-resistant materials.

### 2.3. Experimental Equipment

For the verification of turning with active tool rotation and the evaluation of the monolithic tool design, 90MnCrV8 tool steel was selected for machining (Table 1). The sample dimensions were set to a diameter of 150 mm and a thickness of 20 mm. This steel belongs to the category of high-carbon tool steels, designed for cold-working applications, and exhibits high dimensional stability during heat treatment. Additional properties include high crack resistance, high hardness after heat treatment, moderate toughness, and wear resistance. After the initial machining of the samples, heat treatment was performed to achieve the required hardness levels of the samples. After the heat treatment, consisting of hardening and tempering, the samples reached hardness values of 50, 54, 60, and 63 HRC.

The turning experiments with active tool rotation were conducted on a Hurco VMX 30T (Hurco Company, Indianapolis, IN, USA) three-axis milling center. This milling center has a worktable with dimensions of 1020 × 510 mm and a maximum load capacity of 1000 kg. The tool storage has a capacity of 24 tools, and the spindle has a rated power of 13.5 kW. To conduct the experiments, a machining system equipped with an additional axis was required to enable workpiece rotation. For this purpose, an auxiliary spindle was installed in the working area of the machine. This spindle allows for the adjustment of workpiece rotation speed, ensuring the required cutting speed is achieved. Furthermore, it enables both clockwise and counterclockwise rotation of the workpiece. The auxiliary spindle supports rotation speeds ranging from 110 rpm to 2100 rpm. When setting the required cutting speed, the spindle speed must be adjusted based on the diameter of the machined sample, which is controlled using a potentiometer.

**Figure 4:**
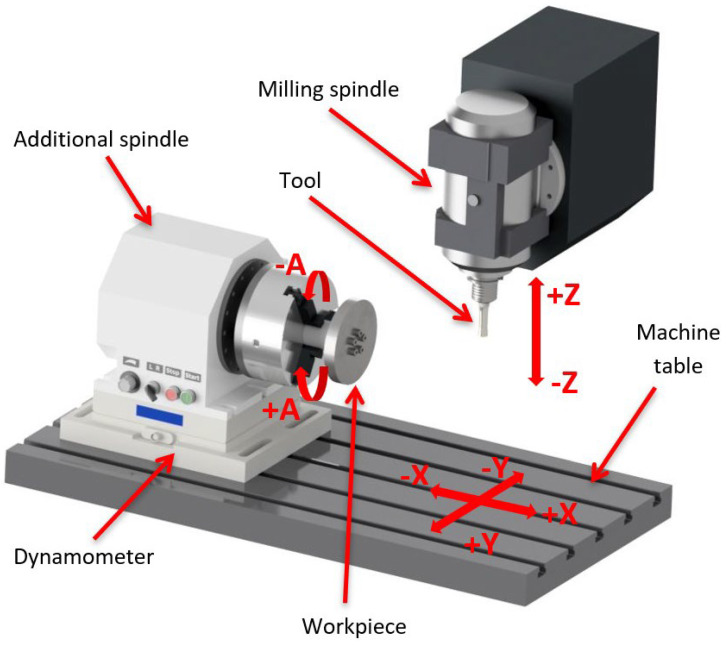
Scheme of machining with active rotation.

The monolithic tool was clamped in the spindle of the machining center (Figure 4). A hydraulic tool holder was selected for clamping, ensuring a firm and stable grip while maintaining the tool alignment with the spindle axis. The Hurco VMX 30t milling center allows for the regulation and adjustment of spindle speed, rotation direction, and cutting speed. Additionally, it provides feed movement along the X, Y, and Z axes. Controlling the movement of the tool in these axes, along with selection of the appropriate rotation speeds for both the tool and the workpiece, enables various machining conditions to be tested depending on the spindle speed and the position of the tool to the workpiece axis. However, the machining center does not support spindle tilting along the B-axis, which means that it is not possible to analyze the effects of tool inclination at an angle.

To measure the dynamics of the cutting process, a three-component dynamometer Kistler 9255A (Kistler Eastern Europe Ltd., Prague, Czech Republic) was placed on the worktable of the machining center. The auxiliary spindle was mounted on the dynamometer, which recorded the components of the cutting force during machining. The auxiliary spindle ensures the rotation of the machined samples. The samples were clamped in the auxiliary spindle using a clamping arbor to provide a secure and stable fixation during turning.

The Alicona InfiniteFocus (Alicona Imaging GmbH, Graz, Austria) optical system was utilized to assess the machined surfaces. This measurement tool enables microscopic evaluation of the coordinates and roughness characteristics of the scanned surfaces. The Alicona Infinite Focus G5 is an optical 3D surface measurement system that uses focus variation technology to provide high-resolution topographical data. When measuring roughness (Rz), the system captures a series of images at different focal planes and reconstructs a detailed 3D surface profile of the machined sample. The device offers high-precision, non-contact measurement, making it particularly useful for evaluating surface roughness in hard materials without introducing additional damage or distortion. The workpiece sample was first secured on the instrument work-bench beneath the confocal microscope. Following proper alignment and illumination of the measured sample, a surface scan was conducted. The scanned surface data were then flattened using the system, and the roughness parameter Rz was evaluated from the acquired data. Rz, also known as the average maximum height of the profile, quantifies surface roughness by considering the vertical distance between the highest peak and lowest valley within a sampling length. It is measured as the average difference between the five highest peaks and five lowest valleys over multiple sampling lengths.

### 2.4. Experimental Design

For the experiments, specific cutting conditions were selected for each sample. A constant tool cutting speed v_t_ = 150 m·min^−1^ was maintained throughout all tests. Additionally, constant values were set for the depth of cut a_p_ = 0.1 mm and the feed rate f = 0.05 mm. The sole variable parameter in the experiments was the cutting speed of the workpiece (v_w_). To accommodate differences in sample hardness, the workpiece cutting speeds were adjusted to 300 m·min^−1^, 500 m·min^−1^, 750 m·min^−1^ and 1085 m·min^−1^. The highest cutting speed of v_w_ = 1085 m·min^−1^ was attained with a 150 mm diameter sample. The overview of the experimental design is provided in Table 2.

## 3. Results

**Figure 5:**
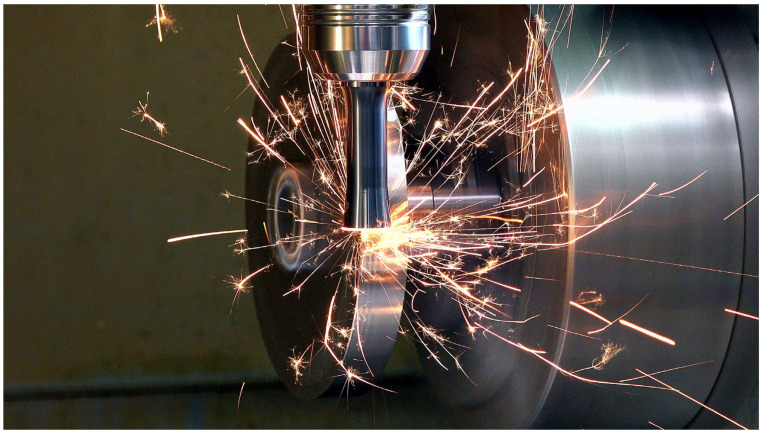
Monolithic rotary tool in the machining process.

The following chapter presents the experimental results of this study. Figure 5 illustrates the experimental setup, showcasing the actively rotary tool engaged in the machining process. The findings detailed below offer insights into the tool’s performance, including cutting forces, wear characteristics, and surface roughness under varying conditions. These results provide a comparative evaluation of the tool with and without a chip divider, highlighting the respective e effects on machining efficiency and workpiece quality.

### 3.1. Function of the Chip Breaker

The chip generated during machining can influence both the machining process and the quality of the machined surface. During turning with active rotation, chip formation depends on several factors, including the tool rotation direction, tool inclination angle, tool rotation speed, and mechanical properties of the machined material (strength, toughness, and structure). Additional influential factors include properties of the cutting material, static and dynamic characteristics of the machine tool, the use of cutting fluid, the geometry of the cutting edge, the type of chip breaker, and specific cutting conditions. In this study, the chips formed during the experiments were analyzed in terms of their shape and size. Detailed images of the chips were taken using an Alicona Infinite Focus G5 confocal microscope (Graz, Austria). Additionally, for tools with a chip breaker, the influence of the chip breaker on the resulting chips was examined.

**Figure 6:**
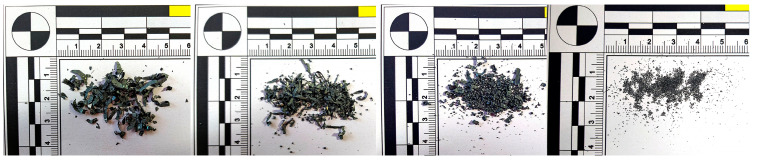
Chip shape at 60 HRC without chip breaker and cutting speeds v_w_: 300 m·min^−1^, 500 m·min^−1^, 750 m·min^−1^, 1085 m·min^−1^.

**Figure 7:**
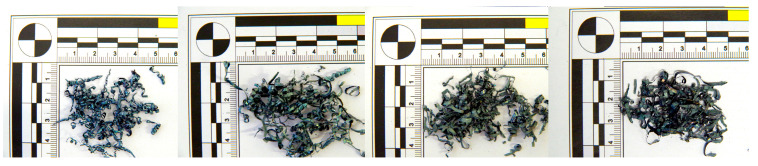
Chip shape at 60 HRC with chip breaker and cutting speeds v_w_: 300 m·min^−1^, 500 m·min^−1^, 750 m·min^−1^, 1085 m·min^−1^.

At higher cutting speeds, the material is sheared more rapidly, resulting in less time for deformation and compression within the chip. The increased speed generates more heat due to greater friction between the tool and the workpiece. This heat is largely carried away by the chips, making them hotter and more malleable. This increased heat and malleability leads to the formation of ball-shaped chips (Figure 6), which have a rounded, spherical appearance rather than the typical segmented or continuous chip forms observed at lower cutting speeds. When applying a tool with a chip breaker (Figure 7), ball chips were not observed, but segmented chips were produced at all v_w_ speeds.

**Figure 8:**
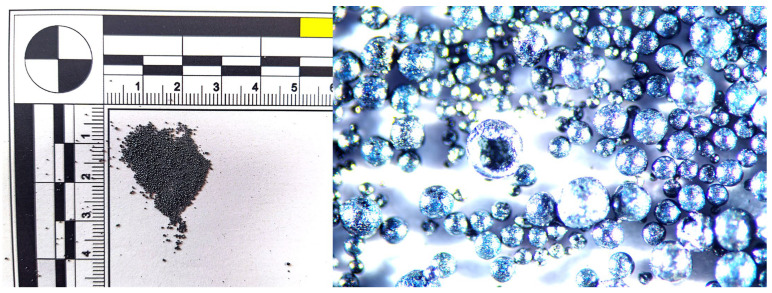
50 HRC, v_w_ = 1085 m·min^−1^, without the chip breaker.

During the analysis of chips generated when using a tool without a chip breaker, it was observed that at a cutting speed of 300 m·min^−1^, the chips exhibited a blue coloration and formed a short helical conical spiral with a thickness ranging from 0.01 to 0.03 mm. The blue color indicates a high temperature at the cutting zone, leading to chip hardening due to heat treatment. As the cutting speed increased to 500 m·min^−1^, the chip shape transformed into a continuous spiral, with a thickness ranging from 0.01 to 0.04 mm. At a cutting speed of 750 m·min^−1^, chip overheating occurred, leading to a color change to gray. Molten deposits formed on the spiral-shaped chips, and chips in the shape of balls also appeared, generated by the melting of the breaking chip due to excessive temperatures. The chip thickness ranged from 0.01 to 0.04 mm. At the highest workpiece cutting speed of 1085 m·min^−1^, the chips took the shape of balls (Figure 8), with diameters ranging from 0.19 to 0.55 mm.

**Figure 9:**
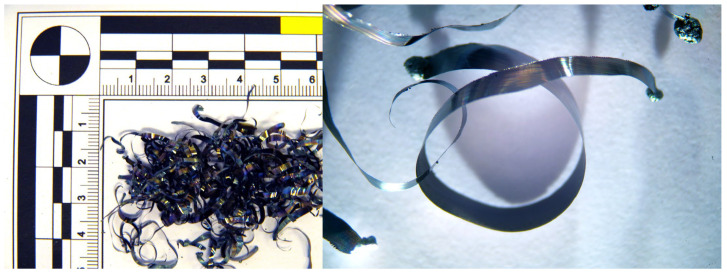
50 HRC, v_w_ = 1085 m·min^−1^, with the chip breaker.

When using the tool with a chip breaker, the following chip formations were observed at different cutting speeds:

- At a cutting speed of 300 m·min^−1^, tubular chips approximately 20 mm long were formed.
- At a cutting speed of 500 m·min^−1^, the chips had a short helical conical shape.
- As the speed increased to 750 m·min^−1^, chips in the shape of ribbons were observed, along with a color change to gray and the formation of molten deposits due to high temperatures. However, in contrast to the tool without a chip breaker, no chips in the shape of balls were observed.
- At the highest cutting speed of 1085 m·min^−1^, the chip breaker functioned effectively, forming short chips in the shape of ribbons with molten deposits at their ends (Figure 9). The absence of chips in the shape of balls indicates a reduction in temperature.

**Figure 10:**
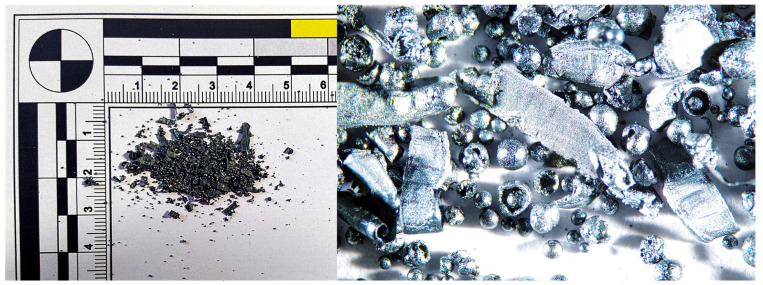
54 HRC, v_w_ = 750 m·min^−1^, without the chip breaker.

**Figure 11:**
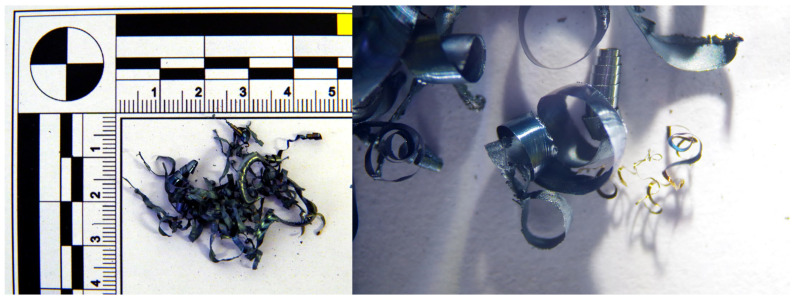
54 HRC, v_w_ = 750 m·min^−1^, with the chip breaker.

For the tool without a chip breaker, at a workpiece cutting speed of 300 m·min^−1^ and a sample hardness of 54 HRC, the chips had a similar shape and color to those observed when machining the 50 HRC sample. As the cutting speed (v_w_) increased to 750 m·min^−1^, molten chips in the shape of balls were formed (Figure 10), ranging in size from 0.54 to 0.86 mm. However, when using a tool with a chip breaker, no molten chips in the shape of balls were observed (Figure 11).

**Figure 12:**
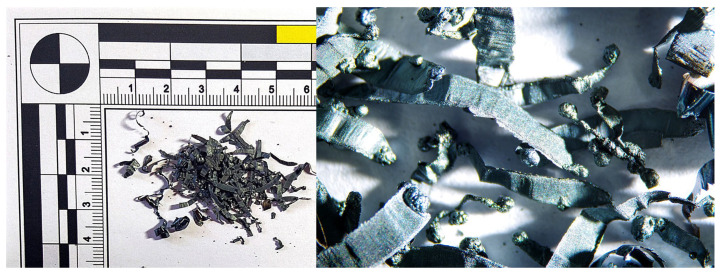
63 HRC, v_w_ = 300 m·min^−1^, without the chip breaker.

When machining materials with hardness levels of 60 HRC and 63 HRC using a tool without a chip breaker, two distinct chip shapes were observed, a combination of short helical chips and chips in the shape of balls. Chip melting into ball shapes occurred even at the lowest cutting speed (v_w_) = 300 m·min^−1^ (Figure 12). The chip thickness ranged from 0.04 to 0.08 mm. At a cutting speed of 500 m·min^−1^, short chips with a thickness of 0.03–0.07 mm formed alongside chips in the shape of balls with diameters ranging from 0.23 to 0.7 mm. At 750 m·min^−1^, chips in the shape of balls were also observed, ranging from 0.2 and 0.5 mm in diameter. At the highest cutting speed of 1085 m·min^−1^, very small elemental chips were formed, along with chips in the shape of balls ranging from 0.15–0.4 mm in diameter.

**Figure 13:**
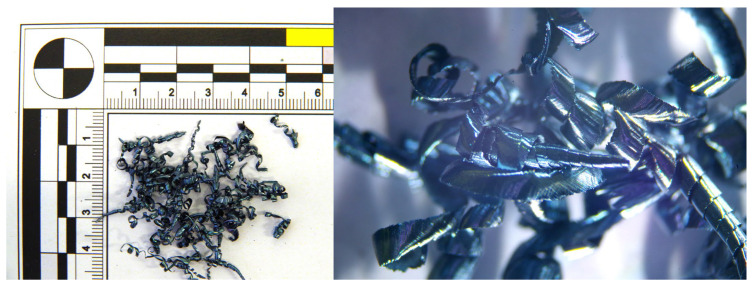
63 HRC, v_w_ = 300 m·min^−1^, with the chip breaker.

When using a tool with a chip breaker, short helical chips were formed at cutting speeds of 300 and 500 m·min^−1^ (Figure 13). At a cutting speed of 750 m·min^−1^, overheating of the generated chips was observed, along with the formation of molten deposits at the chip ends and a color change to gray. The chips became brittle and could be easily crushed by hand. At the highest cutting speed (v_w_) = 1085 m·min^−1^), short chips in the shape of ribbons were formed, partially melting due to the intense heat generated during machining.

### 3.2. The Impact of the Chip Breaker on the Final Cutting Force

**Figure 14:**
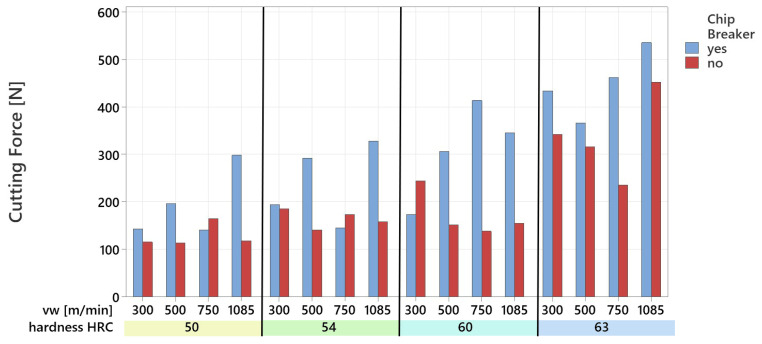
Magnitude of the cutting force in machining with a rotary tool.

The cutting force was measured for all experiments conducted, using both the tool without a chip breaker and the tool with a chip breaker. The entire machining process was recorded, but data from the entry and exit phases of the tool were excluded to eliminate distortions in the results. Based on the measured components of the cutting force, the final cutting force was calculated, and its maximum values are graphically represented (Figure 14).

The cutting force had an increasing trend depending on the hardness of the machined material and the cutting speed v_w_. The lowest cutting force, 113.56 N, was recorded when using a tool without a chip breaker while machining a sample with a hardness of 54 HRC at a cutting speed of 500 m·min^−1^. In contrast, the highest cutting force, 535.89 N, was achieved with a tool equipped with a chip breaker while machining a 63 HRC sample at a cutting speed of 1085 m·min^−1^. For a sample hardness of 60 HRC and a cutting speed of 750 m·min^−1^, the tool with a chip breaker generated three times the cutting force compared to the tool without a chip breaker. Overall, the tool with a chip breaker resulted in higher cutting force values in 81.25% of cases, with an average increase of 1.6 times. The observed increase in cutting force may negatively impact tool wear on the cutting edge and, as a result, reduce tool life.

### 3.3. Surface Roughness After Turning

**Figure 15:**
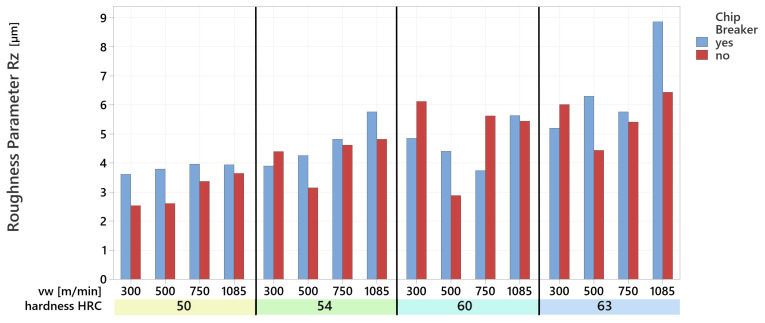
Surface roughness parameter Rz.

The geometry of the cutting tool fundamentally influences the achieved surface roughness. Since this study examines two types of rotary tools, analyzing their impact on surface roughness is essential (Figure 15). The machined surface was analyzed using an Alicona Infinite Focus G5 confocal microscope according to the relevant standard STN EN ISO 21920-1 [38]. The selected roughness parameter Rz was employed as the primary roughness parameter in this study because it provides a more detailed representation of peak-to-valley variations, which is particularly relevant when analyzing surfaces affected by interrupted cutting conditions or chip breaker influences. This metric demonstrates enhanced sensitivity, as the reported Rz value represents the average of Rz measurements from five measured sections. Since Rz measures the average height difference between the highest peaks and deepest valleys over multiple sampling lengths, it offers a better understanding of the actual roughness profile in machining applications where chip formation, tool marks, or irregularities play a significant role.

The measured surface roughness Rz ranged from 2.54 µm to a maximum of 8.87 µm. When machining material with a hardness of 50 HRC using a tool with a chip breaker, the recorded surface roughness Rz varied between 3.62 µm and 3.94 µm, which corresponds to the roughness values typical of finishing operations. This range represents the lowest roughness observed across different cutting speeds (v_w_). As the hardness of the machined material increased, higher Rz values were recorded. However, the change in tool geometry, specifically whether the tool was equipped with a chip breaker and thus introduced an interrupted cut, did not significantly affect the machined surface quality.

### 3.4. Wear of the Cutting Edge of the Rotary Tool

**Figure 16:**
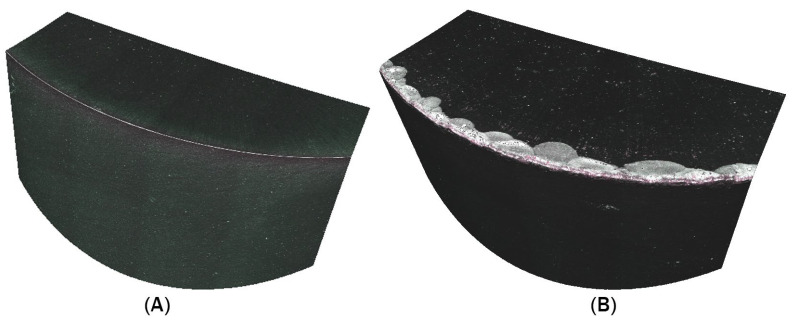
Scanned cutting edge of the tool: (**A**) before turning; (**B**) after turning.

During the machining process, tool wear results from high temperatures generated during material removal, as well as friction between the chip and the tool. The extent of tool wear after the experiments was evaluated using an Alicona Infinite Focus 5G optical microscope. The cutting edges of the rotary tools were scanned both before and after the experiments (Figure 16), and the obtained images were analyzed and compared. Based on this comparison, the extent of tool wear was evaluated.

For the tool without a chip breaker, wear was observed on the rake face, distributed along the entire circumference. Chipping of the cutting edge was evident on the rake face and extended onto the flank face. In addition to edge chipping, abrasive wear was also visible on the flank face.

After machining, the rake face of the tool with a chip breaker was also scanned. Similar to the tool without a chip breaker, the most significant wear appeared on the rake face. Chipping of the cutting edge was again observed, extending to the flank face.

**Figure 17:**
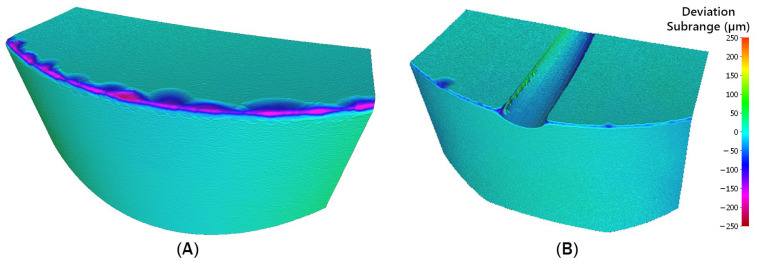
Tool wear comparison: (**A**) tool without chip breaker; (**B**) tool with chip breaker.

When evaluating the tool wear without a chip breaker and analyzing the generated color map (Figure 17), the maximum measured wear was 216.47 µm. In contrast, the maximum wear recorded for the tool with a chip breaker was 133.28 µm. Chipping of the cutting edge in both tools may have been caused by vibrations generated during the experiments. A comparison of the maximum deformation between the tool without a chip breaker and the tool with a chip breaker indicates that the tool with a chip breaker experienced less wear.

## 4. Discussion

The experimental results clearly show that chip formation is strongly influenced by cutting conditions, particularly cutting speed and the presence of a chip breaker. These findings align with previous studies that emphasize the importance of chip control in improving surface quality and extending tool life [39]. As observed, increasing the cutting speed leads to changes in the shape and characteristics of the chips, consistent with chip formation and dynamics theories. At lower speeds (300 m·min^−1^), chips appear as blue and short, indicating relatively lower temperatures and less deformation. As the speed increases, higher temperatures result in the formation of continuous spirals and, at the highest speeds (1085 m·min^−1)^, chips in the shape of balls are formed. When using a tool with a chip breaker, significant differences in chip behaviour were observed, confirming the findings of Kishawy [12]. The chip breaker contributed to chip fragmentation and prevented excessive heating, as evidenced by the absence of chips in the shape of balls at all tested cutting speeds. This observation aligns with other studies demonstrating that chip-breaking elements reduce the contact between the chip and the tool, thereby lowering friction and chip temperature [12,39,40]. High temperatures can cause chip melting, leading to undesirable shapes, such as chips in the shape of balls, which may negatively impact the surface finish of the machined material. Based on these findings, it can be concluded that setting appropriate cutting conditions and incorporating a chip breaker are critical for achieving high surface quality and extending tool life.

The measurement of cutting force in the experiments revealed significant differences between the tools with and without a chip breaker. The results indicate that tool geometry significantly influences cutting conditions and overall machining efficiency. As expected, the increase in cutting force with the hardness of the machined material and cutting speed correlates with theoretical knowledge, indicating that harder materials possess greater resistance to deformation processes during cutting [11,41]. The finding that the tool with a chip breaker exhibited higher cutting force values in 81.25% of cases suggests that the interrupted cutting action caused by the chip breaker increases cutting resistance. The use of a chip breaker geometry can lead to higher cutting forces due to several mechanisms. The chip breaker disrupts the continuous flow of the chip, causing it to break or deflect prematurely. This interrupted chip flow results in periodic increases in cutting resistance, as the material must be repeatedly re-initiated for cutting. Additionally, as the chip breaks into smaller sections, it comes into more frequent contact with the chip breaker or tool surface, leading to increased friction and resistance to chip movement, thereby elevating the overall cutting force. The chip breaker can also alter the material deformation in front of the cutting edge, potentially increasing the energy required to separate the chip, which contributes to higher cutting forces. In summary, while the chip breaker aids in chip control and improves the quality of the machined surface, this benefit comes at the cost of higher cutting forces, which arise from a combination of increased friction, interrupted chip flow, and elevated stress concentration at the cutting edge. Conversely, a reduction in cutting force may indicate lower mechanical stress on the tool, potentially enhancing its life and reducing wear. This observation aligns with hypotheses suggesting that lower cutting forces can extend tool life by minimizing thermal and mechanical loads on the cutting edge [13,42,43]. In general, higher cutting forces pose a potential risk of increased tool wear. Excessive mechanical loads can accelerate damage mechanisms such as edge chipping, abrasive wear, and overall tool degradation, leading to faster tool wear and more frequent replacements. However, as shown in the presented results, the monolithic tool with a chip breaker exhibited lower tool wear, despite experiencing higher cutting forces compared to the tool without a chip breaker. Chip breakers, by design, induce chips to curl and break, reducing the contact length between the hot chip and the tool. This shorter contact time minimizes heat transfer to the tool, mitigating wear mechanisms, like thermal softening and diffusion. Hosokawa et al. [27] illustrate how a chip breaker can induce rapid changes in chip curvature, promoting breakage and thus, potentially, impacting contact length.

The results of this study demonstrate that the geometry of the cutting tool, specifically the presence of a chip-breaking element, has minimal impact on the achieved surface roughness parameter Rz when machining hard materials. Although it was expected that a tool with a chip breaker might degrade surface quality due to interrupted cutting [44], the experimental results contradict this assumption. When machining material with a hardness of 50 HRC, the Rz roughness values ranged from 3.62 µm to 3.94 µm, which are consistent with typical finishing operations. These values did not significantly differ from those achieved with a tool without a chip breaker, indicating that interrupted cutting has little impact on surface quality. Furthermore, the study revealed that the hardness of the machined material has a more pronounced impact on surface roughness than tool geometry. As material hardness increased, Rz values also increased, likely due to changes in material behaviour during machining. Harder materials exhibit greater resistance to cutting, leading to a deterioration in surface quality. These findings suggest that for machining processes requiring low surface roughness, greater attention should be given to selecting an appropriate workpiece material and optimizing cutting parameters such as cutting speed rather than modifying tool geometry.

The evaluation of tool wear after machining provides valuable insights into the impact of the chip-breaking element on tool durability. Wear identified on both the rake and flank faces is a natural consequence of high temperatures and friction occurring during the machining process [43]. However, differences in wear intensity between the tool with a chip breaker and the one without offer important information for optimizing machining processes. Vibrations, identified as a potential factor contributing to the chipping of cutting edge, represent another area for future research. A more detailed analysis of the dynamic behaviour of the cutting process, including vibration measurements, could provide a deeper understanding of their influence on tool wear and support the development of effective strategies to minimize their impact.

Future research should explore optimized chip breaker geometries to balance chip control, cutting forces, and surface quality, minimizing drawbacks while maintaining effective chip segmentation. Conducting a more in-depth investigation into tool wear progression, including high-resolution imaging and quantitative analysis, could provide valuable insights into long-term effects. Furthermore, examining the impact on surface integrity, such as residual stresses, microstructural changes, and hardness variations, would offer a more comprehensive understanding of machined surface quality. Expanding the study to encompass difficult-to-machine materials, like titanium, Inconel, and hardened steels, would help assess the broader applicability of chip breakers. Additionally, further optimization of cutting parameters, including cutting speed, feed rate, and depth of cut, could identify conditions where chip breakers provide the greatest advantage without excessive trade-offs. Lastly, leveraging finite element modeling and computational simulations could predict chip formation behavior, cutting forces, and thermal effects, reducing the need for extensive physical experimentation and accelerating the development of improved machining strategies.

This research contributes to a better understanding of the interactions between cutting tool geometry and the properties of the machined material, which is essential for effective surface quality management in industrial practice.

## 5. Conclusions

The kinematic scheme of turning with active rotation (ADRT) differs from the conventional turning process. In machining with a rotary tool, the rotation of the tool is incorporated into the kinematics, influencing the cutting process. This study focused on identifying a machining process under active rotation and examining chip segmentation when machining hardened materials using a new monolithic tool with a chip breaker. The tool used in this study is a modified version of the patented solution 3-2020, developed by the research team at the Department of Machining and Manufacturing Technology, University of Žilina. The influence of the chip breaker was analyzed in terms of cutting force, surface roughness, tool wear, and chip segmentation functionality. Based on the experimental measurements and obtained data, the following conclusions can be drawn:

- the optimization of cutting conditions and the application of a chip breaker significantly contribute to reducing tool wear, thereby extending tool life;
- controlling chip formation through appropriately selected cutting parameters can reduce thermal load and prevent the formation of undesirable chip shapes, such as molten chips in the shape of balls;
- tools equipped with a chip breaker generally generate higher cutting forces and, while this does not necessarily lead to increased tool wear, further investigation is needed to fully understand its long-term effects;
- chip segmentation through interrupted cutting does not always improve surface roughness and, in some cases, a slight increase in the Rz metric is observed; however, a 3 µm rise in Rz is not necessarily significant, especially considering the typical roughness characteristics of hard materials.

Based on the conducted experimental activities and presented findings, it can be concluded that the proposed chip breaker has a favourable impact on chip segmentation and tool wear reduction. However, a notable drawback is the increase in cutting forces. For this reason, the application of a chip breaker should be considered primarily for machining materials that can withstand higher cutting forces.

## Figures and Tables

**Table 1:** Chemical Composition 90MnCrV8 based on attestation certificate (wt%).

C	Si	Mn	P	S	Cr	V
0.85–0.95	0.1–0.4	1.9–2.1	0.03 max	0.03 max	0.2–0.5	0.05–0.15

**Table 2:** Design of experimental parameters.

Chip Breaker	v_w_ (m·min^−1^)	Hardness HRC
Tool with chip breaker	300	50
	300	54
	300	60
	300	63
	500	50
	500	54
	500	60
	500	63
	750	50
	750	54
	750	60
	750	63
	1085	50
	1085	54
	1085	60
	1085	63
Tool without chip breaker	300	50
	300	54
	300	60
	300	63
	500	50
	500	54
	500	60
	500	63
	750	50
	750	54
	750	60
	750	63
	1085	50
	1085	54
	1085	60
	1085	63

## Data Availability

The original contributions presented in this study are included in the article. Further inquiries can be directed to the corresponding author.
